# Changing clinical perspectives on sex and healthcare disparities in ischaemic heart disease

**DOI:** 10.1016/j.lanepe.2025.101370

**Published:** 2025-08-21

**Authors:** Angela Maas, Edina Cenko, Viola Vaccarino, Irene Göttgens, Maria Bergami, Olivia Manfrini, Lina Badimon, Guiomar Mendieta, Sabine Oertelt-Prigione, Zorana Vasiljevic-Pokracic, Maria Dorobantu, Marija Vavlukis, Bela Merkely, Martha Gulati, Raffaele Bugiardini

**Affiliations:** a Department of Women's Cardiac Health, Radboud University Medical Center, Nijmegen, Netherlands; b Department of Medical and Surgical Sciences, University of Bologna, Bologna, Italy; c Department of Epidemiology, Rollins School of Public Health, Emory University School of Medicine, Atlanta, USA; d Department of Primary and Community Care, Radboud University Medical Center, Nijmegen, Netherlands; e IRCCS Azienda Ospedaliero-Universitaria di Bologna Sant’Orsola Hospital, Bologna, Italy; f University of VIC-UCC, School of Medicine, Barcelona and Cardiovascular Research Foundation for Health Prevention and Innovation, Barcelona, Spain; g Department of Cardiology, Hospital de la Santa Creu i Sant Pau, Barcelona, Spain; h Centro Nacional de Investigaciones Cardiovasculares Carlos III (CNIC), Madrid, Spain; i Sex- and Gender-Sensitive Medicine Department, Medical Faculty OWL, Univeristy of Bielefeld, Bielefeld, Germany; j Medical Faculty, University of Belgrade, Belgrade, Serbia; k Romanian Academy, “Carol Davila” University of Medicine and Pharmacy, Bucharest, Romania; l University Clinic for Cardiology, 1000, Skopje, Republic of North Macedonia; m Faculty of Medicine, Ss. Cyril and Methodius University in Skopje, 1000, Skopje, Republic of North Macedonia; n Semmelweis University Heart and Vascular Center, Budapest, Hungary; o Barbra Streisand Women's Heart Center Smidt Heart Institute, Cedars-Sinai Medical Center Los Angeles CA, USA

**Keywords:** Ischaemic heart disease, Sex disparities, Gender disparities, Risk factors, Acute coronary syndrome

## Abstract

Ischaemic heart disease (IHD) has historically been under-researched in women, leading to significant gaps in understanding sex-specific risk factors and outcomes. To address this issue, The Lancet Regional Health–Europe convened experts from a broad range of countries to evaluate sex-related cardiovascular inequalities and propose recommendations to address these disparities. Despite developing IHD a decade later than men, women experience higher mortality rates. Global Burden of Disease data highlight persistent sex differences in IHD mortality, with women showing higher mortality despite lower prevalence. Factors such as psychosocial stress, reproductive health, and physical inactivity disproportionately impact women's cardiovascular health, while caregiving responsibilities and delayed healthcare access further exacerbate these disparities. There is an urgent need to recognize chest pain symptoms in women and to reduce the time lag between symptom onset and hospital presentation. Addressing these gaps requires targeted public health interventions, expanded research, and improved clinical practices, emphasizing equitable healthcare access and greater inclusion of women in clinical trials. Tailoring treatment guidelines to account for sex differences in outcomes could significantly improve survival rates for women with IHD.

**Key messages**

• Despite advances, significant sex disparities in diagnosis, treatment, and outcomes persist. Women are underdiagnosed, undertreated, and underrepresented in clinical trials, contributing to poorer outcomes.
• Traditional risk factors, such as diabetes, smoking, and hypertension, have a disproportionately higher impact on women. Emerging risk factors like lipoprotein(a) and sex-specific conditions (e.g., pregnancy complications) remain underrecognized in clinical practice.
• Women face unique challenges from socioeconomic factors, caregiving burdens, and gender-specific psychosocial stress, amplifying cardiovascular risks.
• Women with ST-segment elevation myocardial infarction (STEMI) have higher mortality rates due to delayed care, underuse of guideline-recommended treatments, and perhaps biology. Conditions like myocardial infarction with non-obstructive coronary arteries (MINOCA), angina with non-obstructive coronary artery disease (ANOCA), and ischemia with non-obstructive coronary artery disease (INOCA) are more common in women, complicating diagnosis and management, but do not influence outcomes.
• Increased awareness of women's cardiovascular health is essential. Enhanced inclusion of women in clinical trials and the development of sex-specific guidelines are critical. Comprehensive public health initiatives and targeted policies are needed to address disparities.
• Sex-specific considerations should guide risk assessment, treatment strategies, and prevention efforts. Tailored approaches to percutaneous coronary intervention, revascularization, and secondary prevention are necessary to improve outcomes for women.
• Tackling social determinants, ensuring equitable healthcare access, and fostering international collaboration are key to addressing sex disparities in ischaemic heart disease and achieving meaningful progress in women's cardiovascular health.

## Introduction

Throughout much of the 20th century, ischaemic heart disease (IHD) was predominantly studied in men, based on the assumption that it was primarily a male condition. This bias was challenged in 1991 when Bernadine Healy, the first female director of the National Institutes of Health (NIH), launched the Women's Health Initiative. Healy introduced the concept of “Yentl syndrome”,^1^ highlighting the systemic neglect in recognizing and treating IHD in women, whose symptoms often differ from the traditional male-centric presentation of IHD.

Her advocacy led to increased sex-specific research^2^ revealing that women are more likely to suffer from nonobstructive coronary artery disease (CAD)^3^ and often women do not align with the traditional cardiovascular risk profile of men.^4^ Despite some advances, significant gaps persist in understanding why women have higher mortality rates from CAD, a disparity that cannot attributed solely to their older age at presentation.^5^

**Fig. 1:**
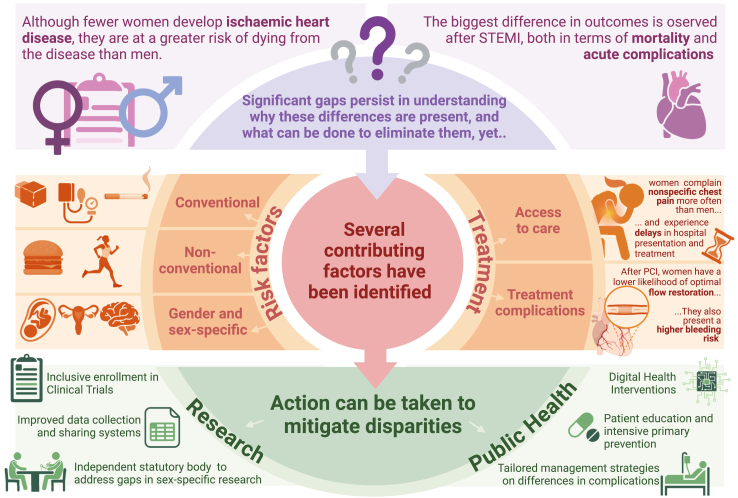
**The evolving clinical landscape of sex and healthcare disparities in ischaemic heart disease**. Created in BioRender.com.

To address these disparities (Fig. 1), The Lancet Regional Health–Europe convened experts from a broad range of countries to evaluate sex-related cardiovascular inequalities. This Series paper explores the current landscape of sex-specific research, emphasizing the need of comparative studies between men and women to refine clinical guidelines.

### Terminology notes

As sex and gender often interact, we used the term “sex” consistently throughout the manuscript to accurately reflect the differences under investigation. However, when discussing risk factors influenced by societal, cultural, and behavioural roles we retained the term “gender” to distinguish these influences.

### Global Burden of Disease data: the ongoing need to address sex disparities in ischaemic heart disease mortality

The Global Burden of Disease (GBD) mortality and population databases provide valuable information about cause-specific deaths, categorized by age group, sex, country, and year (Appendix).^6^ This enables large-scale comparisons, such as those between men and women conducted in the present study. While the data are age-standardised and generally of high quality, there is variability in vital statistics data collected and reported to the GBD across countries. Although this variability may affect direct comparisons of mortality rates between countries, it is less likely to affect differences between men and women within the same country.

To mitigate these limitations, we selected a group of countries all belonging to the EU, as these countries share a politically homogeneous framework. This ensures consistent reporting of population size and mortality data with minimal gaps. Although the most recent year with comprehensive mortality data available for most countries at the time of our analysis was 2022, we decided to use the GBD 2019 data to avoid potential influences on IHD mortality from the COVID-19 pandemic.^7^

**Fig. 2:**
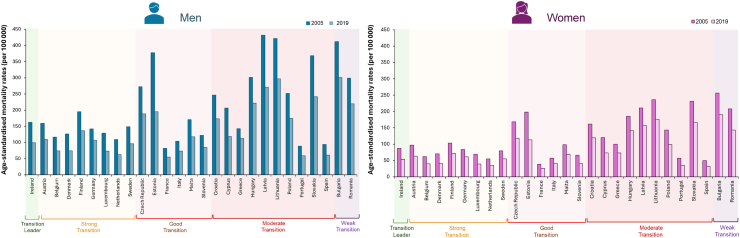
**Trends in age-standardised mortality rates per 100,000 inhabitants for IHD, stratified by country and sex (2005 versus 2019)**. Data driven from the GBD study 2019. Abbreviations: IHD, ischaemic heart disease; GBD, Global Burden of Disease.

**Fig. 3:**
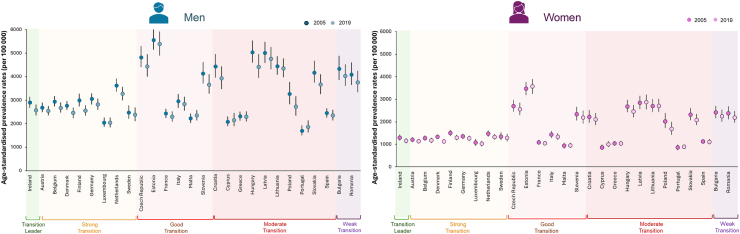
**Trends in age-standardised prevalence rates per 100,000 inhabitants for IHD, stratified by country and sex (2005–2019)**. Data driven from the GBD study 2019. Abbreviations: IHD, ischaemic heart disease; GBD, Global Burden of Disease.

The age-standardised mortality rate for IHD has substantially decreased across the 27 EU countries between 2005 and 2019, in both men and women (Fig. 2 and Table S1). Overall, IHD mortality in EU was approximately twice as high in men than in women in both 2005 (5687 versus 3290 cases per 100,000) and 2019 (3801 versus 2270 cases per 100,000), with some indication that IHD mortality in middle-income countries had declined to a greater extent in men than in women. The declines in age-standardised IHD mortality rates align with the observed trends in the prevalence of the disease (Fig. 3, Table S1). The EU mean age-standardised IHD prevalence rates dropped by 6% between 2005 and 2019. However, this achievement was shared similarly by women and men, leaving in 2019 the prevalence rate in men up to twice as high as that in women (84,648 versus 44,872 cases per 100,000).

**Fig. 4:**
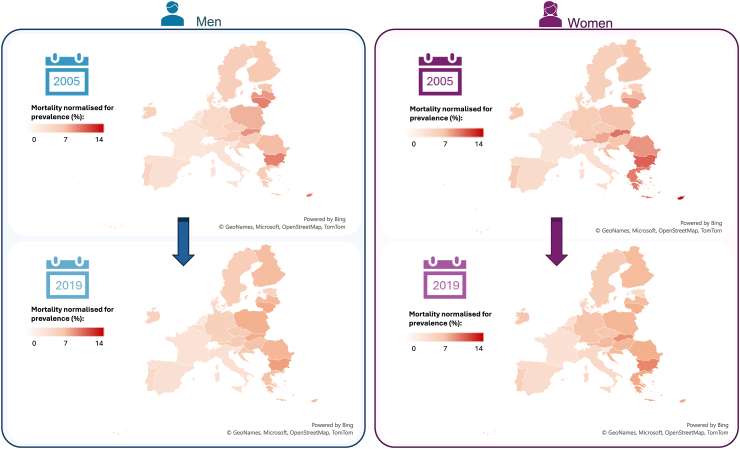
**Trends in mortality from IHD normalised by its prevalence, stratified by country and sex (2005 versus 2019). Data driven from the GBD study 2019**. Abbreviations: IHD, ischaemic heart disease; GBD, Global Burden of Disease. Maps generated using Microsoft Excel and Bing Maps data (© 2024 Microsoft Corporation). Microsoft product screen shots reprinted with permission from Microsoft Corporation.

**Fig. 5:**
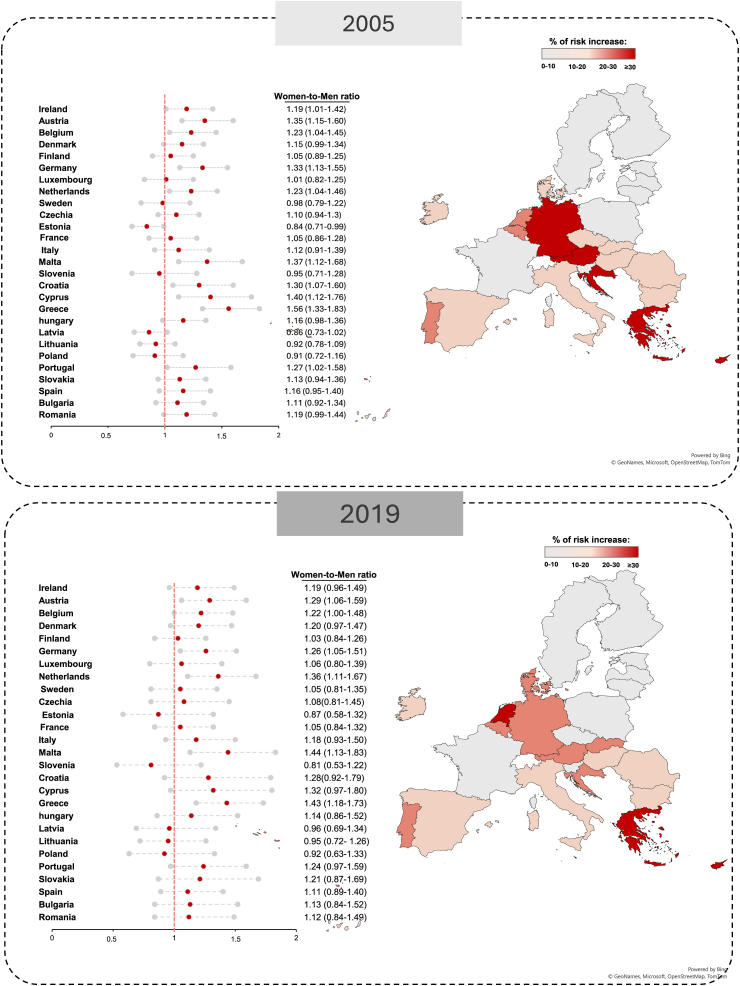
**Changes in women-to-men risk ratio for mortality from IHD normalised by its prevalence, by country, (2005–2019). Data driven from the GBD study 2019**. Abbreviations: IHD, ischaemic heart disease; GBD, Global Burden of Disease. Maps generated using Microsoft Excel and Bing Maps data (© 2024 Microsoft Corporation). Microsoft product screen shots reprinted with permission from Microsoft Corporation.

These findings support the notion that both the development of IHD and its related mortality are more common among men than women within the same age group. They also indicate that reducing mortality rates in both women and men is particularly challenging in regions where IHD prevalence remains high, especially in middle-income countries (transition performance index: moderate and weak performers) (Appendix). However, evaluating IHD mortality normalized to its prevalence (Appendix) provides a different perspective (Fig. 4 and Table S1). The persistently higher age-standardised mortality rates normalized to its prevalence in women compared with men across most countries indicate that although fewer women develop IHD, they are at a greater risk of dying from the disease within the same age group (Fig. 5 and Table S1). Countries that in 2019 showed a remarkable higher mortality in women compared with men include some of Europe's most prosperous countries, such as Germany (4.80% versus 3.80%; risk ratio: 1.26) and Austria (5.56% versus 4.30%; risk ratio, 1.29). This finding suggests that the higher mortality in women is independent of a country's income level and underscores the ongoing need to address sex disparities in IHD mortality.

### Sex differences in risk factors

#### Impact of traditional cardiovascular risk factors on mortality

While both sexes share traditional cardiovascular risk factors, their impact can vary between women and men (Fig. 1). For example, although diabetes is more prevalent in men, it confers a greater relative increase in atherosclerotic cardiovascular disease (CVD) risk in women across all age groups, even though the absolute risk remains higher in men.^8^, ^9^, ^10^, ^11^ A similar trend is observed with smoking, where the relative cardiovascular risk is higher in women compared with men, despite smoking being more prevalent in men.^9^^,^^12^^,^^13^

Data from the Copenhagen City Heart Study and the Copenhagen General Population Study demonstrated that the causal genetic effects of LDL-C on IHD risk are comparable between the sexes.^14^ Similarly, the risk of myocardial infarction rises with increasing body mass index and being overweight or obese for both sexes, with no significant sex differences observed in this relationship.^15^

According to GBD data, hypertension is the leading deadly risk factor in women worldwide and second only to smoking in men.^16^ However, the effect of hypertension on myocardial infarction incidence varies by sex. The hazard ratio (HR) for elevated blood pressure is 1.83 for women compared with men (95% CI, 1.33–2.52), while for stage 1 and stage 2 hypertension, the ratios are approximately 1.5.^15^ These findings prompt discussion about whether normal blood pressure thresholds should be set lower for women than for men.^17^

#### Lipoprotein (a) as a cardiovascular risk factor in women

Lipoprotein(a) [Lp(a)] is a lipoprotein with pro-atherogenic properties that is recognized as an emerging cardiovascular risk factor.^18^ Although Lp(a) plasma levels are genetically determined and one measure is presently considered sufficient in a life-time, different life stages in women, such as menopause, seems to influence Lp(a) levels.^19^ Recent evidence from meta-analyses of randomized controlled trials suggests that hormone replacement therapy (HRT) can reduce Lp(a) concentrations in postmenopausal women by an average of 20–25%.^20^ However, HRT is not recommended for reducing atherosclerotic cardiovascular risk. Further studies with robust designs are needed to clarify the implications of Lp(a) elevation for cardiovascular risk in postmenopausal women.

#### Physical inactivity

The World Health Organization (WHO) estimated that in 2016, insufficient physical activity contributed to 3.2 million deaths globally,^21^ underscoring the importance of addressing physical inactivity as a major public health issue. Despite this, data on sex differences in outcomes related to physical inactivity are scarce. In a large, nationally representative U.S. cohort of 186,724 men and 225,689 women, engaging in regular muscle-strengthening activities, compared with inactivity, was associated with a 11% reduction in cardiovascular risk for men (HR, 0.89; 95% CI, 0.80–0.98) and a 30% reduction for women (HR, 0.70; 95% CI, 0.62–0.78), with a significant interaction between sexes (P_interaction_ = 0.001).^22^

**Fig. 6:**
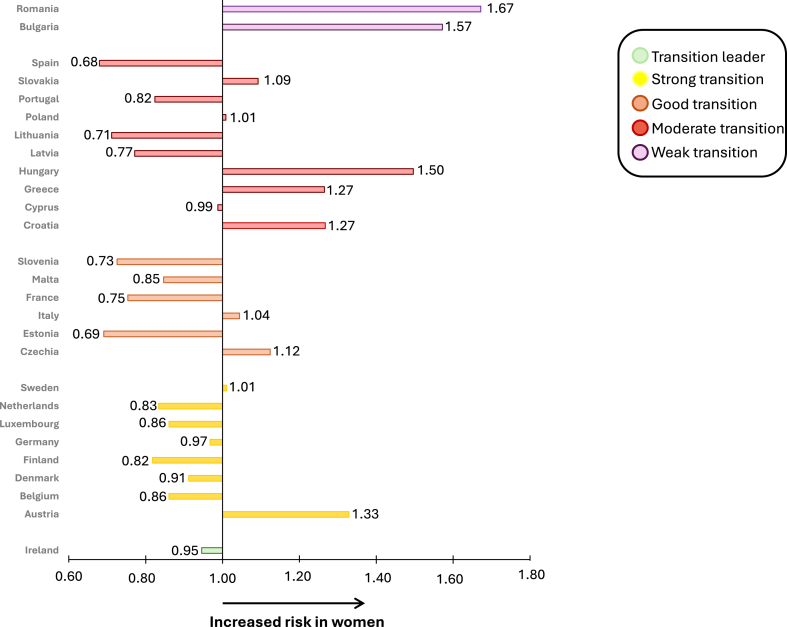
**Women-to-men ratio of death attributable to physical inactivity, stratified by country. Data driven from the GBD study 2019**. Abbreviation: GBD, Global Burden of Disease.

Consistent with these findings, the GBD 2019 study revealed that the total level of daily physical inactivity (less than 3000–4500 MET minutes per week) disproportionately contributed to IHD mortality in women compared with men.^7^ Pronounced differences in women-to-men risk ratios, with over a 40% higher risk for women, were observed in countries with moderate or weak level of economic transition, such as Romania (1.67), Bulgaria (1.57), and Hungary (1.50). In contrast, a lower risk for women, less than 30%, was observed in Spain (0.68). This suggests that as countries undergo economic transition, the disparity in physical inactivity-related mortality between women and men decreases (Fig. 6). In summary, promoting physical activity is a key strategy for reducing IHD mortality, particularly among women in middle-income countries (transition performance index: moderate and weak performers) where the disparity is most pronounced.

#### Unhealthy diet

Several reports from GBD study rank unhealthy diets among the top contributors to disease burden and IHD mortality.^23^ Key dietary risks identified by GBD include high sodium intake and low consumption of whole grains, fruits, nuts, seeds, vegetables, and omega-3 fatty acids. Men generally face a higher burden of CVD death related to dietary factors. In 2019, there were 6.9 million CVD deaths in 2019 attributable to dietary risks, with male accounting for 56.4% of the deaths.^24^ However, the GBD study approach assumes independent effects of each dietary factor, potentially overlooking the more complex interactions between these factors and their combined impact on CVD outcomes.

In contrast, the PURE study took a more integrated approach by creating a comprehensive diet score based on eight food types associated with a lower risk of CVD and mortality: fruits, vegetables, legumes, nuts, fish, dairy, unprocessed red meat, and poultry. A diet score of 4 or lower was more strongly linked to CVD in women than men (HR, 1.17 [95% CI, 1.08–1.26] versus HR, 1.07 [95% CI, 0.99–1.15]; P_interaction_ = 0.0065).^25^

Differences in findings may be due to confounding factors such as geographic region, socioeconomic status, and lifestyle behaviours, which vary between men and women. This underscores the complexity of diet-related risks and the difficulty in unravelling their differing impacts across sexes.

#### Air pollution

Air pollution is a significant risk factor for major noncommunicable diseases, particularly IHD. The WHO estimated that in 2016, ambient air pollution caused 4.2 million deaths globally.^26^ However, research on sex differences in the impact of PM_2.5_ on CVD or IHD mortality remains limited.

A cohort study in China found minimal differences in CVD mortality between sexes, with HRs per 10 μg/m^3^ increase in PM_2.5_ being nearly identical for men (1.17) and women (1.16).^27^ In contrast, data from the GBD 2019 study showed a stronger risk of death from PM_2.5_ exposure, including household air pollution, in men across Europe.^7^ The geographic variation in the effects of PM_2.5_ exposure on cardiovascular outcomes suggests that regional factors, such as pollutant composition and concentration, may play a significant role. Discrepancies in findings may also be explained by differences in exposure assessment methods, pollutant concentrations, and the specific components of air pollution in various regions.

Emerging evidence highlights the role of microplastics and nanoplastics (MNPs) in cardiovascular health. These particles have been shown to trigger consistent inflammatory and immune responses, potentially leading to myocardial injury and elevated cardiac enzymes. However, the lack of standardised sampling and analytical methods for detecting MNP pollution poses challenges to reliably comparing MNP deposition between women and men.^28^ Consequently, further research on air pollution-related CVD or IHD is essential to better understand these disparities and their implications for developing sex-specific public health interventions.

#### Sex-specific risk factors

**Fig. 7:**
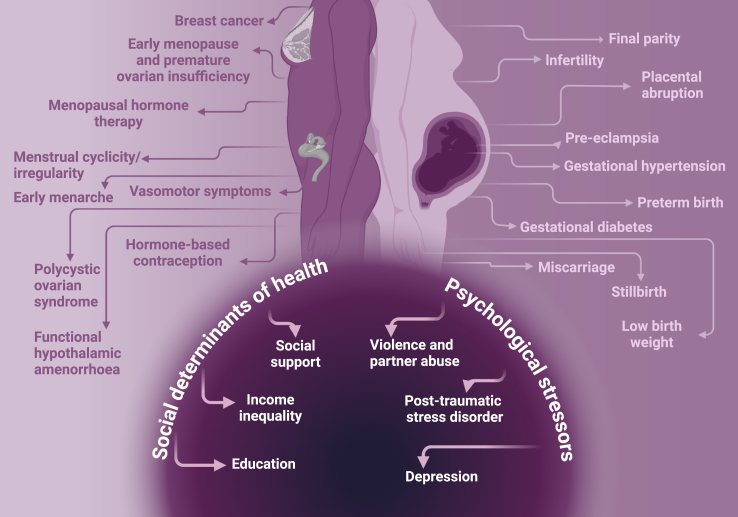
**Sex and gender-specific risk factors for ischaemic heart disease**. Left side of the figure: disorders of the female reproductive system that increase cardiovascular risk. Right side of the figure: pregnancy-related cardiovascular risk factors. Lower panel (dark violet): gender-specific risk factors (social and psychological). Created in BioRender.com.

For women, there are sex-specific risk factors for IHD that remain underrecognized and are not incorporated in our current risk assessment tools. (Fig. 7). These include pregnancy-related outcomes and reproductive issues.^29^ As the traditional risk factors dominate at older age, these sex-specific risk factors are especially important at younger age, when effective prevention should start.

Earlier menarche is a known predictor of cardiovascular risk, as oestrogen provides cardioprotective effects by improving vascular health and reducing arterial stiffness.^30^^,^^31^ Despite their significance, these factors are often overlooked. Physicians should be aware of their long-term cardiovascular impact, and patients should be educated to engage in early prevention strategies. Additional mechanisms linking female reproductive decline to CVD events are detailed in the Appendix.

#### Gender-specific risk factors

Gender-specific risk factors arise from sociocultural influences, such as ethnicity, education, income inequality, and social support, which significantly affect cardiovascular outcomes.^32^^,^^33^ Women are also disproportionately affected by depression, partner violence, and post-traumatic stress disorder. Together, these factors contribute to barriers in healthcare access and increased caregiving stress, both of which are linked to gender roles rather than biological sex.^34^^,^^35^

#### Social determinants of health and psychosocial stress on cardiovascular outcomes

Social determinants of health (SDOH) such as poverty and low education, disproportionately affect women.^36^ A 2017 meta-analysis of 44 studies involving over 22 million people found that while SDOH were linked to IHD in both sexes, factors like lower education, income, and area deprivation posed a significantly higher risk for women compared with men.^37^ However, recent studies have highlighted some convergence in these risks, with household income remaining a stronger predictor of atherosclerotic CVD in women.^38^ These data underscore the complex interplay between socioeconomic factors and cardiovascular health, particularly in women.

#### Intersectionality: minority groups and compound stressors

Black and Hispanic women face additional challenges, including employment inequity, higher rates of obesity, hypertension, and diabetes compared with white women.^39^^,^^40^ Moreover, language barriers and communication issues increase the risk of less guideline-based treatment, especially among Hispanic women in the US who are less likely to have a stable, long-term primary care provider (80.0%) compared with US non-Hispanic white women (91.7%).^41^ Addressing these intersecting barriers is essential to achieve health equity.

#### Psychosocial stress as an amplifier of SDOH impacts

Psychosocial stress acts as a critical mediator of the impact of SDOH on cardiovascular outcomes. Stressors such as caregiving burdens, exposure to violence, and economic insecurity contribute to an increased risk of mood and anxiety disorders, which in turn amplify cardiovascular risk through neurobiological and physiological pathways. Women are more likely to experience a distinctive burden of psychosocial adversities, are more prone to stress-related mood and anxiety disorders, and more vulnerable to adverse cardiovascular effects resulting from stressors.^42^ Stress-related activation of the sympathetic nervous system and the hypothalamic–pituitary–adrenal axis influences cardiovascular physiology through elevated cortisol levels, increased blood pressure, and systemic inflammation.^43^ As gonadal hormones play a critical role in modulating the body's stress response, women may exhibit heightened vulnerability to stress-induced effects, contributing to sex-specific differences in cardiovascular outcomes.^44^ Additional mechanisms linking stress and CVD are detailed in the Appendix and summarized in Panel 1.

Panel 1 **Additional mechanisms linking stress and cardiovascular disease.**

1. **Impact of** **psychosocial** **stress on** **cardiovascular** **disease:**
  o Psychosocial stress contributes to elevated cortisol levels, increased blood pressure, and systemic inflammation, adversely affecting cardiovascular physiology.^42^, ^43^
  o Women are disproportionately affected, experiencing higher rates of stress-related mood disorders and unique stressors like caregiving and domestic violence.^45^
2. **Sex-specific** **stress** **responses:**
  o Women exhibit heightened inflammatory responses to stress, reduced glucocorticoid sensitivity, and increased platelet aggregation, amplifying cardiovascular disease risk.^42^
3. **Stress and** **microvascular** **dysfunction:**
  o Women demonstrate greater peripheral microvascular vasoconstriction under stress, linked to reduced coronary microvascular flow.^46^
  o This dysfunction is implicated in conditions disproportionately affecting women, such as:
    • Ischemia with non obstructive coronary artery disease (INOCA).^45^
    • Mental stress-induced myocardial ischemia.^45^, ^47^
    • Stress-induced (Takotsubo) cardiomyopathy.^42^
4. **Cumulative** **stressors:**
  o Childhood adversity (e.g., sexual abuse) induces “biological embedding,” altering nervous, endocrine, and immune systems, and increasing lifetime cardiovascular disease risk.^48^
  o Unpaid caregiving, undertaken by over 80% of women globally, is a chronic stressor linked to hypertension and inflammation.^49^

### Sex differences in presentation and outcomes

#### Sex differences in stable and unstable IHD

IHD encompasses various clinical presentations and associated mortality risks. While earlier studies suggested women faced higher risks after all types of acute coronary syndromes (ACS), recent findings show that sex differences in outcomes are most pronounced in ST-segment elevation myocardial infarction (STEMI). Given the considerable differences in treatment strategies and outcomes between STEMI and non-ST-segment elevation (NSTE)-ACS, it may be useful to analyse them separately. This section summarizes important women-specific aspects for clinical presentation of IHD and the implications of clinical presentation for sex differences in outcomes after percutaneous coronary intervention (PCI) treatment (Fig. 1, Panels 2 and 3).

Panel 2 **Key points and recommendations for clinical presentation of ischaemic heart disease (IHD).**

**ST-segment elevation myocardial infarction (STEMI)**

• **Key** **points:**
  o Women with STEMI have higher in-hospital and 1-year all-cause mortality compared with men.
  o Women present later to the hospital after symptom onset than men
  o Women are at a higher risk of developing acute heart failure upon hospital admission for STEMI.
• **Recommendations:**
  o Increase awareness among women about the importance of early hospital presentation.
  o Ensure timely diagnosis and treatment by avoiding the use of the term “atypical” to describe chest pain.
  o Tailor treatment guidelines to consider sex-specific differences in the relationship between delay to treatment and mortality
  o Implement evidence-based strategies to improve outcomes in women with STEMI, including prompt reperfusion therapy.
  o Implement primary prevention therapies to reduces the risk of STEMI as initial manifestation of cardiovascular disease.

**Non-ST-segment elevation myocardial infarction (NSTEMI)**

• **Key** **points:**
  o Data on sex differences in outcomes for non-ST-segment elevation acute coronary syndromes are mixed.
  o Women with NSTEMI have similar rates of acute heart failure as men.
• **Recommendations:**
  o Conduct further research to clarify potential sex-specific factors influencing NSTEMI outcomes.
  o Promote equitable use of diagnostic investigations and guideline-recommended therapies for women.
  o Address potential biases in symptom evaluation and risk perception among healthcare providers.

**Myocardial infarction with non-obstructive coronary arteries (MINOCA)**

• **Key** **points:**
  o MINOCA is more common in women than in men.
  o In-hospital mortality rates for MINOCA are similar between women and men.
  o Long-term outcomes show no significant sex differences in all-cause mortality.
• **Recommendations:**
  o Recognize MINOCA as a distinct clinical entity requiring specific diagnostic and therapeutic approaches.
  o Ensure that both men and women receive appropriate follow-up and management for MINOCA.
  o Investigate underlying mechanisms to better understand if sex-specific differences in MINOCA outcomes exist.

**Ischemia with non-obstructive coronary artery disease (INOCA)**

• **Key** **points:**
  o Symptoms and signs of INOCA are more common in women than men.
  o Current trials have not yet reported on quality of life and outcomes for INOCA patients.
  o Prior studies have been insufficient in addressing sex differences in INOCA outcomes.
• **Recommendations:**
  o Improve diagnostic evaluation for women and men presenting with ischemia and nonobstructive CAD.
  o Conduct research to determine if sex differences in outcomes exist for INOCA.
  o Develop management strategies tailored for both men and women with INOCA

Panel 3 **Implications of clinical presentation for sex differences in outcomes after percutaneous coronary intervention (PCI) treatment**

**Sex differences in outcomes after PCI**

• **Key** **points:**
  o Increased risk for women in ACS: Women undergoing PCI are at increased risk of myocardial infarction, and ischemia-driven target lesion revascularization compared with men in meta-analyses including patients predominantly with ACS.
  o No significant sex differences in chronic coronary syndromes : Meta-analyses including patients predominantly with chronic coronary syndromes found no significant sex differences in outcomes.
• **Recommendations:**
  o Consider sex-specific factors in ACS: Incorporate sex-specific factors in PCI risk assessment and management for ACS.
  o Investigate complications in ACS: Study sex differences in periprocedural complications during PCI in ACS.
  o Tailor interventions in ACS: Customize antithrombotic and antiplatelet therapy, thrombectomy, and embolic protection based on sex and clinical presentation.

**Sex-related differences in bleeding risk after PCI**

• **Key** **points:**
  o Women have an excess bleeding risk due to a higher incidence of procedural vascular complications.
  o Women are more often at high bleeding risk (HBR) than men according to the Academic Research Consortium (ARC) criteria.
• **Recommendations:**
  o Implement tailored strategies to mitigate bleeding risk in women undergoing PCI.
  o Conduct further research to determine the impact of reducing bleeding events on overall outcomes.
  o Ensure accurate assessment of bleeding risk and use appropriate preventive measures in women.

#### Recognizing chest pain in women

Women often present later than men after symptom onset, delaying timely reperfusion therapy. While chest pain is the most common symptom in both sexes (over 90%),^50^^,^^51^ women may also experience associated symptoms like diaphoresis, dyspnoea, and nausea, which can obscure the diagnosis.^52^ To address this challenge, the 2021 Chest Pain Guideline from the American Heart Association and the American College of Cardiology recommend no longer using the term “atypical” to describe chest pain in women,^53^ as it may imply “noncardiac” and ultimately results in less intensive care and delayed treatment.

#### Outcomes in STEMI

A meta-analysis of 35 studies (18,555 women and 49,981 men) with STEMI treated with primary PCI found women had nearly 1.5 times the adjusted risk of in-hospital mortality compared with men.^54^ These disparities persisted at 30 days, even after adjusting for age, comorbidities, angiographic disease severity, primary PCI, and medications used at admission.^51^ Although the reasons for these disparities in STEMI outcomes are not fully understood, common explanations for worse outcomes in women include delayed hospital presentation possibly due to less common symptoms and undertreatment with guideline-recommended therapies.^55^

#### Impact of presentation delays on mortality in women with STEMI

The “time is muscle” hypothesis would imply that both men and women, presenting with similar delays, would have similar adverse outcomes.^56^ However, an analysis of the International Survey of Acute Coronary Syndromes (ISACS) registry suggests that the optimal timing for treatment may vary for men and women.^57^ This study stratified patients by prehospital delay in hours and found that the disparity in outcomes became more pronounced as the delay extended, with mortality rates in women being 1.29 times higher for delays within 2 h and 1.84 times higher for delays within 4 h, when compared with men. Similarly, a large analysis from the French Metaregistry confirmed that longer ischaemic times in women are predominantly due to patient delay in seeking care, with limited opportunities for improvement in the medical care process in such cases.^58^

These findings suggest that women are more vulnerable to severe ischemia, indicating a need to reconsider guidelines for STEMI reperfusion care. Applying identical time-to-treatment approaches to both men and women, without considering potential sex-specific differences in the impact of delays on myocardial injury and function, could result in suboptimal outcomes for women. There is an urgent need to investigate this matter further.

#### Non-ST-segment elevation acute coronary syndromes

The data on sex differences in outcomes for NSTE-ACS are mixed. The National Registry of Myocardial Infarction study, covering 361,429 patients from 1057 US hospitals, found that younger women had a 15–20% higher adjusted mortality rate than younger men, regardless of myocardial infarction type, while older women showed no mortality differences compared with their male counterparts.^59^ Conversely, a study using the National Inpatient Sample database reported that women with NSTEMI had 10% lower odds of in-hospital mortality than men after adjusting for age, PCI use, and comorbidities.^60^ Similar findings were reported in an analysis of the Thrombolysis In Myocardial Infarction (TIMI) clinical trial database, which showed that women with NSTE-ACS had higher 30-day mortality in unadjusted models but a 16% lower risk after multivariable adjustment.^61^ Nonetheless, women with NSTE-ACS in the TIMI study remained undertreated with guideline-directed medical therapies.

Overall, these mixed results highlight that while sex disparities in outcomes exist, they are complex and influenced by various still unknown factors. Women may be at both higher and lower risk depending on the context,^62^ suggesting that sex-specific considerations are relevant and necessary for improving NSTE-ACS treatment and outcomes.

#### Sex differences in mortality associated with heart failure after ACS

Most studies of the association between sex and ACS prognosis have focused on mortality, with data on complications being scarce and often conflicting. Clinical outcomes in patients who present with ACS complicated by heart failure (ACS-HF) are of concern because these patients show markedly higher short- and long-term mortality than those without ACS-HF. Studies conducted by the ISACS investigators provide important insights into sex differences in the risk of acute HF following different types of ACS.^63^, ^64^, ^65^ Women are at a higher relative risk of developing acute HF on hospital admission for STEMI than men (33.7% versus 29.0%). By contrast, the risk for acute HF in NSTE-ACS patients is similar between women (25.6%) and men (25.1%), indicating that the sex-related risk difference is less pronounced in this type of ACS.^64^ These studies raise questions about the underlying causes of these sex differences and may help to explain the higher mortality observed in women after STEMI.

#### Myocardial infarction and non-obstructive coronary arteries (MINOCA)

There is no difference in all-cause mortality between sexes in patients presenting with MINOCA.^66^ Among 322,523 myocardial infarction patients in the ACTION Registry-GWTG, MINOCA was more common in women than men (10.5% versus 3.4%), but in-hospital mortality was similar (1.1% versus 1.0%).^67^ These outcomes align with the ISACS registry findings, where 30-day mortality was 1.5% in women and 1.9% in men.^9^ In the SWEDEHEART registry, all-cause mortality at four-year follow-up was 13.4%, with no significant differences between women and men.^68^ Thus, it is reasonable to conclude that MINOCA does not explain the large sex disparities in outcomes seen in IHD.

#### Considerations for chronic coronary syndrome in women

Women have a slightly higher prevalence of chronic coronary syndromes than men, with a pooled sex ratio of 1.20.^69^ However, women have a lower incidence of test-positive cases of angina.^70^ This diagnostic gap may be partly attributed to the higher prevalence of non-obstructive CAD in women, as well as the perception among physicians of lower cardiovascular risk in women presenting with chest pain during testing.

Importantly, women with test-positive angina had higher standardised mortality ratios for coronary heart disease than men up to age 75 years. For those aged 55–64 years, the ratio was 4.69 (95% CI, 3.60–6.11) in women compared with 2.40 (95% CI, 2.11–2.73) in men. The rate of coronary revascularization was also higher in men than women due to more obstructive CAD. However, adjusting for receipt of revascularization during the first year of follow-up did not affect the sex differences in coronary event rates.^70^

Similarly, in the ISCHEMIA trial, women had less obstructive CAD and, therefore, fewer revascularizations. Notably, there were no sex differences in the primary major adverse outcome (HR women versus men, 0.93; 95% CI, 0.77–1.13)^71^ not only despite lower revascularization rates but also despite lower risk factor goal attainment in women.

In summary, these observations demonstrate inequities in the management of women presenting with chest pain. Chronic coronary syndrome in women is often underestimated. Research is needed to determine the best ways to identify and manage chest pain in women.

#### Angina with non-obstructive coronary artery disease (ANOCA)

Up to 40% of patients undergoing cardiac catheterization for suspected obstructive CAD have no evidence of significant epicardial disease, a condition termed ANOCA.^72^ Despite normal angiograms, these patients remain at increased risk of cardiovascular events and often experience reduced quality of life due to persistent symptoms, frequent hospitalizations, and repeat procedure. The diagnosis and management of ANOCA present significant challenges, as physicians and patients alike struggle to reconcile ongoing angina-like symptoms with the absence of obstructive findings. This uncertainty can perpetuate symptoms and complicate care.

The aetiology of ANOCA is multifactorial, with coronary vasomotor abnormalities, such as microvascular dysfunction and epicardial vasospasm, increasingly recognized as key contributors. These mechanisms underscore the need for targeted diagnostic and management strategies. Epicardial coronary artery constriction, symptom reproduction, and ischaemic electrocardiographic changes during acetylcholine testing have been proposed as criteria for diagnosing epicardial or microvascular spasm.^4^^,^^73^ However, these criteria must be interpreted cautiously as recent data suggest that microvascular dysfunction, defined as an index of microcirculatory resistance ≥25 during acetylcholine testing, is present in only 10% of patients with nonobstructive CAD.^74^

Further complicating the understanding of ANOCA is the uncertain prevalence of vasospastic angina, with conflicting evidence on whether it is more common in men or women.^73^^,^^75^ These controversies highlight the complexity of ANOCA and the ongoing need for research to refine diagnostic criteria.

#### Ischemia with non-obstructive coronary artery disease (INOCA)

The low diagnostic yield of coronary angiography applies even to patients experiencing typical angina pectoris, with non-invasive stress tests suggesting inducible myocardial ischemia, commonly referred to as INOCA. INOCA is more common in women than men.^4^^,^^76^ In the ISCHEMIA trial, women presented more frequently with INOCA than men (34.4% versus 11.3%).^77^ However, the reported prevalence of INOCA in ISCHEMIA was lower compared with the PROMISE trial, which found potentially ischaemic symptoms in 53% of women and 46% of men.^78^ This discrepancy likely arises from differences in inclusion criteria and study design between the two trials, as the ISCHEMIA trial focused on patients with more well-defined ischaemic symptoms and objective evidence of ischemia, whereas PROMISE had broader inclusion criteria.

Neither the ISCHEMIA nor the PROMISE trials have reported relationships between quality of life and outcomes for these patients, nor do they provide data on angina symptoms in excluded participants with obstructive CAD. As a result, it remains unclear whether there are sex differences in outcomes for patients with angina and INOCA. This underscores the need for further research to determine if such sex differences exist and, if they do, to develop appropriate management strategies for both men and women with INOCA.

### Sex differences in treatment

#### Sex differences in outcomes after PCI

A large meta-analysis pooling patient-level data across 21 stent trials, including 32,877 patients predominantly (65%) with ACS, found that female sex was independently associated at 5 years with an increased risk of major adverse cardiac events (HR, 1.14; 95% CI, 1.01–1.30), myocardial infarction (HR, 1.24; 95% CI, 1.01–1.53), and ischemia-driven target lesion revascularization (HR, 1.23; 95% CI, 1.05–1.44) compared with men.^79^ In contrast, a prespecified subgroup analysis of the GLOBAL LEADERS trial, involving nearly 16,000 patients with predominantly (53%) stable CAD undergoing PCI, suggested that at one-year, women were at comparable risk of death, myocardial infarction, stent thrombosis, and any revascularization after accounting for clinical covariates compared with men, but at a higher risk of bleeding and haemorrhagic stroke following PCI.^80^

This apparent paradox highlights the complexity of analysing all-comer populations undergoing contemporary PCI. Potential explanations for contrasting data include differences in the type of ACS and the proportion of patients with stable versus unstable coronary syndromes. This hypothesis is supported by prior work. Pre-specified subset analyses from the COURAGE trial suggested that women with chronic coronary syndrome derive at least comparable benefit from PCI compared with men.^81^ In contrast, a meta-analysis of more than 500,000 patients with STEMI undergoing primary PCI reported a higher risk for in-hospital mortality (OR, 1.91; 95% CI, 1.84–1.99) in women compared with men.^82^ Additionally, an angiographic analysis revealed that women with STEMI were more likely to experience suboptimal TIMI blood flow (0–2) despite minimal residual diameter stenosis (<25%). This remained true even after adjusting for baseline differences, including time from symptom onset to hospital presentation, suggesting underlying sex differences in coronary physiology or response to PCI.^83^

These findings suggest that sex and clinical presentation may influence post-PCI outcomes. Further research is needed to explore how choice of upstream and intraprocedural antithrombotic and antiplatelet therapy, percutaneous thrombectomy, and distal embolic protection can be optimized to improve outcomes for women undergoing PCI.

#### Sex differences in bleeding risk after PCI

Women face an elevated bleeding risk during PCI, driven by a higher incidence of procedural vascular complications and a higher bleeding risk according to the Academic Research Consortium criteria.^84^ However, there are no evidence showing that reducing bleeding events improves outcomes.^85^ These findings highlight the need for tailored strategies to mitigate bleeding risk in women undergoing PCI. Despite this, bleeding risk alone does not appear to be the primary factor driving sex disparities in outcomes of PCI.

#### Sex disparities in cardiosurgical myocardial revascularization

Women consistently experience worse outcomes after isolated coronary artery bypass graft (CABG) compared with men, including higher mortality and increased rates of major adverse postoperative events, such as stroke and myocardial infarction. A meta-analysis of 966,492 patients reported that women were 66% more likely to die within 30-day post-CABG, with disparities persisting at one- and five-year follow-up.^86^ Remarkably, this sex difference in outcomes appears to diminish with age and is inversely associated with it.^87^^,^^88^ Potential contributors of these differences are driven by factors such as incomplete revascularization,^89^ coronary spasm,^53^ and the number of arterial grafts used during surgery^88^ Improving outcomes for women undergoing CABG, particularly younger women, requires further research and targeted efforts to address these disparities.

#### Sex differences in optimal medical therapy

Studies reveal substantial underprescription of evidence-based therapies for women with coronary heart disease. In Europe, EUROASPIRE IV^90^ and INTERASPIRE^91^ surveys revealed highlight poorer risk factor control and significant geographic and sex disparities in secondary prevention. Only 21.8% of women received optimal medical therapy compared to 41.4% of men in the Australian Health Survey.^92^ Similar patterns are also seen in NHANES.^93^ Among cardiovascular drugs, statin therapy is particularly underutilized in women, as shown by the analysis of the Department of Veterans Affairs^94^ and the Medical Expenditure Panel Survey.^95^ Women remain less likely to receive optimal therapy after myocardial infarction across all age groups.^96^ Governments and healthcare institutions must implement targeted interventions to address these disparities and enhance secondary prevention efforts.

### Public health initiatives and community engagement

Addressing sex disparities in IHD outcomes requires comprehensive public health initiatives and active community engagement (Fig. 1). Increasing awareness that CVD is the leading cause of death among women is critical to preventing poorer outcomes, as women tend to downplay their symptoms and delay seeking treatment.^57^ Programs like Go Red for Women by the American Heart Association, The Heart Truth by the NHLBI, and the European Commission campaigns have contributed to raising awareness (Panel 4).

Panel 4 **Overview of current public health efforts to address cardiovascular health disparities**

**European** **initiatives,** **policy,** **advocacy and** **research**
  • **EU4Health** **programme**: Launched in response to the COVID-19 pandemic, this program aims to strengthen health systems and improve health outcomes, including addressing cardiovascular health disparities. It focuses on reducing health inequalities and promoting healthier lifestyles.
  • **Gender-specific** **research** **projects**: The European Union's (EU) research and innovation programs, Horizon 2020 and Horizon Europe, fund numerous projects focusing on gender differences in health. These projects aim to improve understanding of cardiovascular diseases in women and develop effective prevention and treatment strategies
  • **European Heart Network (EHN)**: A network of heart foundations and Non-Governmental Organizations across Europe, EHN advocates for policies to prevent cardiovascular disease and reduce health inequalities. They work on policy recommendations, public awareness campaigns, and research initiatives.
  • **European Commission's** **health** **policies:** The European Commission has included gender equality in health as a priority in its policies. This includes funding for research on gender differences in health, promoting gender-sensitive healthcare practices, and addressing social determinants of health that disproportionately affect women
  • **National** **prevention** **strategies:** Various EU states have developed national prevention strategies that include specific measures to address cardiovascular health disparities in women. These strategies focus on promoting healthy lifestyles, improving access to care, and reducing socioeconomic disparities (e.g., **The Netherlands- National Prevention Agreement:** This policy framework emphasizes preventive care and healthy lifestyle promotion, with specific measures to address cardiovascular health disparities among women; **Italy- Application and dissemination of gender medicine in the National Health System**: In early 2018, Italy approved a law aimed at integrating gender-specific medicine into the National Health System. The law's objective is to ensure that all medical specialties acknowledge and address sex and gender differences, which is crucial for delivering personalized and effective healthcare.; **UK–National Health Service (NHS) Long Term Plan**: The NHS has set out specific strategies to tackle heart disease and stroke, particularly among disadvantaged groups. This includes improving access to preventive services and addressing social determinants of health.)
  • **Sex and Gender Equity in Research (SAGER) guidelines**: developed by the European Association of Science Editors (EASE) to encourage a more systematic approach to the reporting of sex and gender in research across disciplines.
  • **EU Manifesto for Women's Health 2024:** launched by the European Institute of Women's Health (EIWH) calls on the EU to commit to the reduction of health inequalities and provide equitable health for all women, through the provision of an EU Strategy for Women's Health. Such a strategy would ensure that women's health remains a policy and research priority, and ensure that women's voices and needs are embedded in all EU policies.
**Healthcare System Interventions**
  • **Gender-sensitive** **training for** **healthcare** **providers**: Many European countries have implemented training programs for healthcare providers to recognize and address gender-specific symptoms of heart disease. This includes educational modules on the differences in presentation and risk factors between men and women
  • **Enhanced** **access to** **preventive** **care**: Efforts to improve access to preventive services for women, such as screenings for heart disease risk factors and lifestyle intervention programs, are being implemented across the EU
**US Initiatives and** **programs**
  • **Million Hearts® Initiative**: Launched by the CDC and CMS, this initiative aims to prevent 1 million heart attacks and strokes within five years by promoting cardiovascular health through public education, policy changes, and healthcare system improvements.
  • **The Heart Truth® Campaign**: An initiative by the National Heart, Lung, and Blood Institute (NHLBI) that focuses on raising awareness about heart disease in women, particularly targeting women of colour who are at higher risk.
  • **WISEWOMAN (Well-Integrated Screening and Evaluation for WOMen Across the Nation)**: This program provides low-income, uninsured, and underinsured women with chronic disease risk factor screening, lifestyle programs, and referral services to reduce cardiovascular disease. Integrating innovative, evidence-based strategies for heart disease and stroke prevention within health care systems and communities.
  • **The Inclusion of Women and Minorities as Subjects in Clinical Research**: National Institute of Health (NIH) Revitalization Act of 1993, PL 103-43, signed into law on June 10, 1993, directed the NIH to establish guidelines for inclusion of women and minorities in clinical research.
**Public** **awareness and** **education** **campaigns**
  • **Heart** **health in** **women** **campaigns:** National heart foundations across Europe run campaigns to educate women about heart disease, emphasizing the importance of recognizing symptoms early and making lifestyle changes to reduce risk
  • **European Society of Cardiology (ESC)- Women in** **cardiology** **programme:** This program aims to support female cardiologists and address gender disparities within the profession. It includes mentorship, networking opportunities, and advocacy for gender-specific research in cardiovascular health
  • **ESC** **patient** **websites:** multiple languages campaigns aimed at raising awareness about heart disease including and practical advice to prevent cardiovascular disease, manage their health more effectively, understand, the signs, symptoms and treatments of diseases and live longer, more active lives. (https://www.escardio.org/The-ESC/Advocacy/esc-patient-websites).
  • **Go Red for Women**: An American Heart Association (AHA) campaign aimed at raising awareness about heart disease in women and promoting healthy lifestyle choices.
  • **Check. Change. Control.®**: Another AHA initiative that focuses on helping individuals track their blood pressure and make lifestyle changes to manage hypertension, a key risk factor for cardiovascular disease.

Despite progress, recent data are concerning: by 2019, only 44% of women recognized heart disease as their greatest health threat, a decline from 56% in 2012, with young women showing the greatest drop in awareness.^97^ An urgent redoubling of public health initiatives focused on women's health is required to reverse these trends. In this context, digital health interventions, such as telemedicine, mobile health, and remote monitoring, hold promise for promoting cardiovascular health among women (Appendix).

Greater inclusion of women in clinical trials is another pressing need. While women are better represented in trials for hypertension and atrial fibrillation, they remain significantly underrepresented in coronary heart disease research.^98^ The participation-to-prevalence ratio (PPR) is approximately −18% for ACS trials, reflecting a critical gap in understanding sex-specific outcomes.^98^ Concerns about trial safety, distrust in the healthcare system, and sociocultural barriers often hinder participation.^99^

Practical steps should be undertaken to develop new strategies to achieve optimal recruitment so that the proportion of trial participants is representative of the proportion with the disease in the population. Panel 5 outlines some potential solutions to mitigate the underrepresentation of women in cardiovascular research trials. Community leaders and healthcare providers can motivate women to engage in research, while multicentric registries, focusing on pregnancy-related conditions and IHD, can fill critical research gaps. Regular feedback from community members can refine and improve these efforts.

Panel 5 **Proposed solutions to mitigate the underrepresentation of women in cardiovascular trials.**

**Patient-related solutions**

**Outreach** **initiatives**
  o Use social media, internet, and television to inform women about ongoing cardiovascular (CV) trials.
**Engagement of** **healthcare** **providers**
  o Encourage primary care doctors and heart specialists to share information about CV trials with their female patients.
**Overcoming** **systemic** **barriers**
  o Offer telehealth for follow-up visits to reduce travel needs for patients.
  o Increase the number of trial locations in local communities to make it easier for women to participate.
**Support** **services**
  o Provide childcare and transportation to and from trial centres to facilitate participation.
**Patient-centred** **communication**
  o Have open discussions between research staff and patients about joining trials, possibly involving the patient's primary care provider or cardiologist

**Clinical care solutions**

**Referral** **systems**
  o Set up call centres and coordinators at specialized care centres to help local doctors refer patients to trials.
**Provider** **education**
  o Teach healthcare providers about the importance of focusing on women's specific needs and building trust with patients.
**Promoting** **diversity and** **inclusion**
  o Create a welcoming environment in healthcare institutions that values diversity at all levels.

**Government/Funding solutions**

**Mandated** **participation** **rates**
  o Require government and industry-funded trials to include a fair number of women participants.
**Increased** **funding for** **women-focused** **clinial** **trials**
  o Advocate for legislation to increase funding for clinical trials that focus on women's cardiovascular health.
**Mandated** **analysis and** **reporting**
  o Mandate that government-funded clinical trials to report results separately for men and women, and systematically document reasons for participants drop-out.

**Research/Investigational solutions**

**Diverse study design and steering committee**
  o Actively include and increase representation of women in the steering committee
  o Create mentorship opportunities for women to prepare them for leadership roles in future committees and trials.
  o Ensure that the trial design considers the specific needs and perspectives of women.
  o Design sex-specific analysis
**Diverse** **research** **staff** **recruitment**
  o Hire research staff from diverse ethnic and gender backgrounds to improve trial inclusivity.
**Diversity and** **inclusion** **officer**
  o Appoint an officer to ensure a diverse research environment and promote inclusivity at each trial site.

### Policy recommendations

This Series paper highlights significant concerns regarding the insufficient attention given to mitigating sex disparities in IHD mortality. We recommend a comprehensive strategy, including improved data collection systems to enable accurate analysis of sex differences in IHD mortality and outcomes. Targeted interventions should focus on regions where women have higher mortality despite lower prevalence rates.

A radical reform, consisting of the creation of an independent statutory body at the national level to address gaps in sex-specific research, would be necessary. Mandating sex-specific reporting in government and industry-funded trials would ensure equitable treatment and resources. Regular reports on sex disparities in IHD outcomes should be published to maintain transparency and drive continuous improvement. Finally, investments to reduce variability in secondary prevention both between countries and among individuals, are essential to promote equity in global efforts to reduce the burden of IHD.

### Clinical practice recommendations

Accumulating evidence indicates that women, particularly women with STEMI, may represent a unique high-risk group requiring special attention. The main mechanism causing excess mortality in women compared with men appears to be the longer prehospital delay experienced by women. There is an urgent need to enhance diagnostic evaluation for women presenting with chest pain to ensure timely and appropriate care.^51^^,^^72^ Raising awareness among women and educating healthcare providers about the heightened risk of mortality with STEMI in women may help mitigate adverse outcomes.

Intensive primary prevention therapy could shift the clinical presentation of IHD from STEMI to NSTE-ACS.^65^^,^^100^^,^^101^ Since sex disparities in IHD outcomes are largely driven by the higher occurrence of STEMI in women, it is reasonable to propose that more aggressive use of statins in primary prevention, especially in women, could reduce this gap.

Incorporation of sex-specific factors in the risk assessment and management of patients undergoing PCI, particularly for ACS must be considered. Tailoring antithrombotic therapy, thrombectomy, and embolic protection based on sex and clinical presentation may improve outcomes for women. Implementing strategies to reduce bleeding risk in women undergoing PCI, ensuring accurate assessment and preventive measures, is warranted.

## Conclusions

With this Series paper, we have provided a robust, evidence-based, and diverse set of recommendations for strategies to mitigate the sex gap in mortality from IHD. Nevertheless, many knowledge gaps remain. Structural racism and social determinants, such as limited access to healthcare, healthy foods, and education, play critical roles in these disparities. Addressing these factors on a global scale is necessary, as their relative importance varies across countries.

Despite improvements, sex disparities in IHD mortality persist, even in high-income countries, though these gaps have narrowed between 2005 and 2019. Most countries still report unfavourable IHD outcomes for women, but not all, highlighting that a gradual and meaningful change is achievable. Substantial progress in improving cardiovascular health outcomes for women can only be achieved through concerted global efforts by researchers, healthcare professionals, policymakers, and key stakeholders.

## Contributors

AM and RB conceived the study. AM, EC and RB and planned the methodology, that was reviewed by all authors. AM, EC, VV, IG, LB, and RB conducted the search. AM, EC, VV, IG, LB, and RB reviewed the literature and conducted the analysis. AM, VV and RB wrote the original draft of the paper. EC, and MB produced the manuscript figures and tables. AM, EC, VV, IG, MB, OM, LB, GM, OS, MD, MV, BM, MG and RB participated in the interpretation, reviewing and editing drafts and critically revised the manuscript for intellectual content.

AM, EC; VV, OM, LB, ZVP, MD, MV, BM, MG and RB are part of The Lancet Regional Health-Europe commission on inequalities and disparities in cardiovascular health.

## Editor note

The Lancet Group takes a neutral position with respect to territorial claims in published maps and institutional affiliations.

## Declaration of interests

Professor Bela Merkely declares having received institutional grants from: Boehringer Ingelheim, Duke Clinical Institut, Eli Lilly, Novartis. He declares having received consulting fees from Boehringer Ingelheim, Daiichi Sankyo and Novartis, as well as speaker fees from Boehringer Ingelheim and Novartis. He declares having the following leadership or fiduciary role in other board, society, committee or advocacy group, paid or unpaid: Rector (academic leader) of Semmelweis University, Director and Chair of the Heart and Vascular Center of Semmelweis University, National leader of Librexia Program, National leader of New Amsterdam trial, National leader of DAPA ACT HF-TIMI 68 trial, National leader of MIRACLE trial, National leader of FINEARTS-HF trial, National leader of REALIZE-K trial, National leader of SOS-AMI trial, National leader of DELIVER trial, National leader of GARDEN-TIMI 74 trial, National leader of ENDEAVOR trial, National leader of EMPACT-MI trial, National leader of CARDINAL-HF trial.

Professor Marija Vavlukis declares having received payment or honoraria for lectures, presentations, speakers' bureaus, manuscript writing or educational events from KRKA Pharma, Phizer, Alkaloid. She declares having received support for attending meetings by Phizer, Professor Martha Gulati declares having received grants from the National Heart, Lung, and Blood Institutes nos. N01-HV-068161, N01-HV-068162, N01-HV-068163, N01-HV-068164, grants U01 HL064829, U01 HL649141, U01 HL649241, K23 HL105787, K23 HL125941, K23 HL127262, K23HL151867, T32 HL069751, R01 HL090957, R03 AG032631, R01 HL146158, R01 HL146158-04S1, R01 HL124649, R01 HL153500, U54 AG065141, General Clinical Research Center grant MO1-RR00425 from the National Center for Research Resources, the National Center for Advancing Translational Sciences Grant UL1TR000124, Department of Defense grant PR161603 (CDMRP-DoD), and grants from the Gustavus and Louis Pfeiffer Research Foundation, Danville, NJ, The Women's Guild of Cedars-Sinai Medical Center, Los Angeles, CA, The Ladies Hospital Aid Society of Western Pennsylvania, Pittsburgh, PA, and QMED, Inc., Laurence Harbor, NJ, the Edythe L. Broad and the Constance Austin Women's Heart Research Fellowships, Cedars-Sinai Medical Center, Los Angeles, CA, the Barbra Streisand Women's Cardiovascular Research and Education Program, Cedars-Sinai Medical Center, Los Angeles, CA, The Society for Women's Health Research, Washington, D.C., the Linda Joy Pollin Women's Heart Health Program, the Erika Glazer Women's Heart Health Project, the Adelson Family Foundation, Cedars-Sinai Medical Center, Los Angeles, CA, Robert NA. Winn Diversity in Clinical Trials Career Development Award (Winn CDA), and the Anita Dann Friedman Endowment in Women's Cardiovascular Medicine & Research. This work is solely the responsibility of the authors and does not necessarily represent the official views of the National Heart, Lung, and Blood Institute, the National Institutes of Health, or the U.S. Department of Health and Human Services. She also declares being Immediate Past President of the ASPC.

Other authors declare no conflicts of interest.

**Search strategy and selection criteria.**

We identified relevant articles using a systematic search of the MEDLINE database through PubMed from Jan 1, 1990 to Dec 31, 2024. The search strategy included keywords and their combinations, such as “Women”, “Gender” “Female sex”, Sex disparities”, “Sex differences”, “Coronary heart disease mortality”, “Ischaemic heart disease mortality”, “Cardiovascular health”, “Cardiovascular prevention”, “Cardiovascular prevention” and “Cardiovascular health promotion”. We also reviewed registry studies, and used mortality and population data from individual European Union (EU) states provided by the Global Burden of Disease study accessible databases to incorporate the most comprehensive data available.^6^
