# Supplementary material for: Changing clinical perspectives on sex and healthcare disparities in ischaemic heart disease
Source: Lancet Reg Health Eur. 2025 Aug 21;56:101370. doi: 10.1016/j.lanepe.2025.101370 (PMC12587326; doi:10.1016/j.lanepe.2025.101370)
Supplement: Supplementary Appendix [file mmc1.pdf]

Supplementary appendix

Changing Clinical Perspectives on Sex and Healthcare Disparities in Ischemic Heart Disease

Supplementary appendix

Ischaemic heart disease mortality normalized to its prevalence 2
Measures of national income 2
Sex-specific reproductive risk factors - impact in later life cardiovascular health 2
Perinatal depression 4
Psychosocial stress and cardiovascular outcomes 4
The unique burden of psychosocial stressors in women 4
The role of caregiving stress 5
Childhood adversity and "biological embedding" 5
Sex differences in stress response 5
Biological mechanisms linking psychosocial stress and cardiovascular outcomes 5
Stress and microvascular dysfunction 6
Technology and digital health 6
Table S1. 2005-2019 IHD age-standardised prevalence rate, mortality rate, mortality rates normalized for prevalence, and risk ratio women to men in individuals of all ages, per 100,000 inhabitants. Data from GBD 2019 8
REFERENCES 12

Ischaemic heart disease mortality normalized to its prevalence

The mortality from ischaemic heart disease (IHD) normalized for prevalence is a value that determines whether a country, a region or a demographic group of patients has a higher or lower mortality for the disease under examination. Women-to-men rate ratios for death from IHD in relation to the prevalence of IHD per 100,000 inhabitants were also calculated. A rate ratio of 1.0 indicates equal rates in the two groups, a rate ratio greater than 1.0 indicates a relative increased risk for women, and a rate ratio less than 1.0 indicates a relative decreased risk for women.

Confidence Interval (CI) of the ratios was calculated by means of the formula CI= ln(x1/x2) +- 1.96*SE (logRR), where the standard error was extrapolated with the delta method.

Measures of national income

There are several categories of national income definition. The Transitions Performance Index (TPI) is the one that is used most commonly in the European Commission.[1] The TPI identifies four income ranking areas: (1) transition leader, (2) strong transition, (3) good transition, (4) moderate transition, and (5) weak transition. Throughout the manuscript, the term "high-income countries" represents a composite of leaders and strong or good performers, while the term "middle-income countries" refers to moderate and weak performers.

Sex-specific reproductive risk factors - impact in later life cardiovascular health

Research efforts focused on women's cardiovascular health have significantly increased during the past decades, but still, female-specific cardiovascular disease (CVD) risk factors remain understudied and largely unrecognized. The sex-specific reproductive risk factors identified are premature menopause, gestational diabetes, hypertensive disorders of pregnancy, preterm delivery, polycystic ovary syndrome, and systemic inflammatory and autoimmune disorders[2]. Reproductive history including miscarriage and stillbirth and a short reproductive lifespan have been also associated with increased risk of CVD in postmenopausal women [3-5]. Hormonal disorders in women of reproductive age and these reproductive-age factors are associated with higher risk of developing

chronic cardiometabolic diseases after menopause but possibly as well in the years preceding menopause.

Amongst women, the onset of cardiovascular events generally occurs later in life relative to men. Considering sex-specific reproductive risk factors occur early in a woman's life, an opportunity for unique risk factor identification and early intervention exists. Yet, this opportunity is often overlooked.

An evolving number of sex-specific factors to aid in the recognition and assessment of women's CVD risk have emerged[6-8] but in addition to the traditional risk factors, reproductive age-related factors should also be considered CVD risk factors and/or CVD risk enhancers (**Figure 6 in Main Text**). Sex-specific reproductive risks need to be incorporated into risk scores, or risk assessment algorithms given the recent data showing their association with adverse CVD evolution over women's lifespan.

Our understanding of female-specific CVD risk factors is continuously growing. Recent research has shown genetic links between cardiometabolic disorders and sex-specific risks[9], as well as proteomic profiles that are associated with diseases in peri- and postmenopausal women identifying over 50 protein markers and pathways associated with CVD. Genetic and proteomic studies during the reproductive years may help to detect and predict CVD later in life.

Research efforts should also be directed at providing a better understanding of the physiology of female reproductive decline considering it as a modifiable event, because preserving age-related hormonal functions is key to provide increased life choices and possibly lengthen health span. Estrogen confers cardio-protective effects by improving circulation and vascular health, maintaining oxidative balance, and reducing fibrosis and arterial stiffness in the vasculature[10].

Perinatal depression

Perinatal depression (PND) is another underrecognized factor contributing to maternal cardiovascular risk. Research indicates that PND increases maternal CVD risk by 36%. These findings suggest that including reproductive history—such as PND and other stress-related reproductive conditions—in CVD risk assessments could improve early prevention strategies tailored to women[11]. Therefore, reproductive history including PND should be considered in a woman's CVD risk assessment[11].

Psychosocial stress and cardiovascular outcomes

Psychosocial stress has consistently been associated with negative CVD outcomes in both healthy individuals and those with preexisting CVD.[12] Women are more likely to experience a distinctive burden of psychosocial adversities, are more prone to stress-related mood and anxiety disorders, and more vulnerable to adverse cardiovascular effects resulting from stressors.[13] Stress-related activation of the sympathetic nervous system and the hypothalamic-pituitary-adrenal axis influences cardiovascular physiology through elevated cortisol levels, increased blood pressure, and systemic inflammation[14]. As gonadal hormones play a critical role in modulating the body's stress response, women may exhibit heightened vulnerability to stress-induced effects, contributing to sex-specific differences in cardiovascular outcomes[15].

The unique burden of psychosocial stressors in women

Women, particularly young and middle-aged, face a unique burden of psychosocial stressors that increase their risk of CVD later in life. Over one in four ever-partnered women under age 50 experiences physical, sexual, or psychosocial abuse by a partner. Such experiences have been associated with a 30-50% higher risk of CVD, diabetes, and all-cause mortality[16].[17,18] Furthermore, domestic violence frequently contributes to mental health conditions such as depression and posttraumatic stress disorder (PTSD), both of which are established risk factors for CVD

The role of caregiving stress

In addition to domestic violence, women disproportionately shoulder the burden of unpaid caregiving, comprising over 80% of informal caregivers globally. Informal caregiving is recognized as a chronic stressor, linked to increased prevalence of CVD risk markers, including hypertension and elevated inflammation[19]. These cumulative stressors exacerbate women's cardiovascular vulnerability over time

Childhood adversity and "biological embedding"

Childhood adversity, particularly sexual abuse is more common in girls than boys and is linked to a 50% higher incidence of CVD events in adulthood. This type of trauma induces long-term changes in the nervous, endocrine, and immune systems, a process known as "biological embedding," [20], that increases the risk of chronic conditions. Studies suggest that the association between childhood trauma and CVD is stronger in women than in men.[21]

Sex differences in stress response

Women exhibit a more pronounced inflammatory response to acute mental stress compared with men, with decreased glucocorticoid sensitivity, leading to more prolonged inflammation and increased platelet aggregation and higher CVD risk[22,23]. These sex-based differences in stress response underscore the need for tailored interventions to mitigate their cardiovascular impact.

Biological mechanisms linking psychosocial stress and cardiovascular outcomes

Psychosocial stress has consistently been associated with negative CVD outcomes in both healthy individuals and those with pre-existing CVD.[12] Women are more likely to experience a distinctive burden of psychosocial adversities, are more prone to stress-related mood and anxiety disorders, and more vulnerable to adverse cardiovascular effects resulting from stressors.[13] Stress-related activation of the sympathetic nervous system and the hypothalamic-pituitary-adrenal axis influences cardiovascular physiology through elevated cortisol levels, increased blood pressure, and

systemic inflammation[14]. As gonadal hormones play a critical role in modulating the body's stress response, women may exhibit heightened vulnerability to stress-induced effects, contributing to sex-specific differences in cardiovascular outcomes[15].

Stress and microvascular dysfunction

Women with coronary disease exhibit more pronounced peripheral microvascular vasoconstriction both at rest and following mental stress compared with men, a response linked to decreased microvascular flow at the coronary level.[24] Stress-induced microvascular constriction likely contributes to cardiac conditions that occur disproportionally in women and where microvascular function is thought to play a role. One is non-obstructive coronary artery disease (INOCA); in fact, women with this condition show enhanced peripheral vasoconstriction in response to mental stress.[25] Other conditions include mental stress-induced myocardial ischemia and stress-induced (Takotsubo) cardiomyopathy.[25,26] Similar to inflammation, microvascular changes with mental stress have been associated with adverse CVD events in women but not in men.[27,28]

Technology and digital health

Digital health interventions (DHIs), including telemedicine, mobile health, remote monitoring, and wearables, are essential for improving adherence and supporting lifestyle changes that promote cardiovascular health (CVH)[29]. These interventions enhance CVH by improving healthcare visit adherence, facilitating blood pressure monitoring, supporting weight control, and promoting physical activity and healthy behaviors[30]. This is particularly important for women at various life stages, including pregnancy and the postpartum period[31].

DHIs such as mHealth and eHealth services offer personalized digital health technologies that track and collect biometric and self-reported health data over time. This approach could significantly increase the availability of women-specific cardiovascular data, addressing the gender data gap[32]. The information gathered from these technologies can be used to develop targeted,

gender-sensitive interventions and to validate artificial intelligence (AI)-based screening and risk prediction models[33]. By incorporating diverse, real-world data, these models will improve in both accuracy and relevance for women's CVH.

Despite their potential, inequitable access to DHIs has been highlighted since the COVID-19 pandemic, particularly for women who are older, from ethnic minority groups, non-English speaking, low-income, or living in areas without broadband Internet access[34]. These disparities point to structural barriers in universal access to DHIs.

As DHIs become more widespread, formal evaluations by trusted organizations, such as cardiovascular professional societies, will be essential to ensure quality and effectiveness. Expanding access to DHIs and increasing the representation of women and minority groups in product development, clinical research, and health service deployment are crucial steps in preventing the widening of CVH disparities and ensuring that all populations benefit from digital health advances.

Table S1. 2005-2019 IHD age-standardised prevalence rate, mortality rate, mortality rates normalized for prevalence, and risk ratio women to men in individuals of all ages, per 100,000 inhabitants. Data from GBD 2019

Country	Age-standardised prevalence rate of IHD (100,000)				Age-standardized mortality rate for IHD per 100,000 inhabitants				Mortality rates normalized for prevalence %				Women to men ratio	
	2005		2019		2005		2019		2005		2019		2005	2019
	Men	Women	Men	Women	Men	Women	Men	Women	Men	Women	Men	Women		
**Transition leader**														
**Ireland**	2902 (2674 - 3145)	1297 (1164 - 1432)	2578 (2357- 2823)	1155 (1035 - 1282)	163.08 (152.75 - 169.84)	87.08 (76.15 - 93.05)	99.6 (86.85 - 106.76)	53.27 (44.39 - 59.12)	5.62 (5.1 - 6.19)	6.72 (5.83 - 7.74)	3.86 (3.38 - 4.42)	4.61 (3.87 - 5.49)	1.19 (1.01 - 1.42)	1.19 (0.96 - 1.49)
**Strong transition**														
**Austria**	2687 (2495 - 2898)	1206 (1104 - 1313)	2548 (2356 - 2758)	1136 (1036 - 1243)	159.41 (148.14 - 168.63)	96.91 (84.85 - 104.94)	109.48 (99.67 - 118.2)	63.13 (53.31 - 70.88)	5.93 (5.37 - 6.55)	8.04 (7.02 - 9.2)	4.3 (3.83 - 4.82)	5.56 (4.71 - 6.57)	1.35 (1.15 - 1.6)	1.29 (1.06 - 1.59)
**Belgium**	2939 (2736 - 3171)	1274 (1163 - 1401)	2676 (2476 - 2907)	1175 (1062 - 1293)	116.43 (109.16 - 121.53)	62.01 (54.14 - 67.18)	74.25 (67.85 - 78.75)	39.61 (33.83 - 43.78)	3.96 (3.62 - 4.34)	4.87 (4.23 - 5.6)	2.78 (2.49 - 3.09)	3.37 (2.87 - 3.96)	1.23 (1.04 - 1.45)	1.22 (1 - 1.48)
**Denmark**	2766 (2587 - 2967)	1335 (1239 - 1438)	2462 (2234 - 2692)	1126 (1019 - 1240)	126.67 (118.16 - 132.8)	70.45 (61.65 - 75.74)	73.88 (67.15 - 78.96)	40.49 (34.2 - 44.91)	4.58 (4.19 - 5.01)	5.28 (4.66 - 5.98)	3 (2.65 - 3.39)	3.59 (3.05 - 4.24)	1.15 (0.99 - 1.34)	1.2 (0.97 - 1.47)
**Finland**	2999 (2734 - 3279)	1503 (1370 - 1649)	2556 (2344 - 2782)	1295 (1175 - 1431)	195.02 (183.11 - 202.63)	103.09 (89.74 - 110.26)	137.34 (126.23 - 146)	71.39 (59.84 - 79.14)	6.5 (5.86 - 7.21)	6.86 (5.99 - 7.86)	5.37 (4.8 - 6.01)	5.51 (4.66 - 6.52)	1.05 (0.89 - 1.25)	1.03 (0.84 - 1.26)
**Germany**	3058 (2819 - 3291)	1353 (1244 - 1465)	2822 (2590 - 3064)	1270 (1153 - 1386)	142.62 (133.21 - 148.63)	83.77 (73.44 - 89.51)	107.22 (98.58 - 113.44)	60.76 (52.6 - 66.38)	4.66 (4.24 - 5.12)	6.19 (5.46 - 7.02)	3.8 (3.41 - 4.24)	4.79 (4.14 - 5.54)	1.33 (1.13 - 1.55)	1.26 (1.05 - 1.51)
**Luxembourg**	2053 (1873 - 2261)	1075 (932 - 1237)	2047 (1846 - 2261)	1026 (905 - 1163)	129.42 (120.59 - 137.21)	68.64 (60.59 - 74.41)	73.33 (63.53 - 82.44)	38.91 (31.47 - 45.32)	6.31 (5.63 - 7.07)	6.39 (5.37 - 7.6)	3.58 (3.04 - 4.22)	3.79 (3.05 - 4.71)	1.01 (0.82 - 1.25)	1.06 (0.8 - 1.39)
**Netherlands**	3631 (3367 - 3926)	1476 (1341 - 1612)	3275 (2993 - 3568)	1327 (1205 - 1462)	109.12 (101.47 - 114.46)	54.77 (48 - 59.02)	63 (57.05 - 67.53)	34.74 (29.55 - 38.5)	3.01 (2.73 - 3.31)	3.71 (3.24 - 4.25)	1.92 (1.7 - 2.17)	2.62 (2.23 - 3.07)	1.23 (1.04 - 1.46)	1.36 (1.11 - 1.67)

**Sweden**	2476 (2205 - 2787)	1350 (1190 - 1533)	2384 (2112 - 2695)	1290 (1130 - 1466)	148.75 (138.8 - 156.25)	79.64 (69.21 - 86.51)	96.04 (87.92 - 103.22)	54.41 (45.64 - 62.8)	6.01 (5.27 - 6.85)	5.9 (4.99 - 6.97)	4.03 (3.48 - 4.66)	4.22 (3.44 - 5.18)	0.98 (0.79 - 1.22)	1.05 (0.81 - 1.35)
**Good transition**														
**Czech Republic**	4820 (4402 - 5296)	2693 (2448 - 2980)	4432 (4001 - 4953)	2562 (2298 - 2861)	272.62 (256.18 - 282.2)	168.29 (152.1 - 177.75)	188.86 (158.64 - 221.57)	117.97 (95.3 - 138.33)	5.66 (5.1 - 6.28)	6.25 (5.52 - 7.08)	4.26 (3.49 - 5.19)	4.6 (3.72 - 5.7)	1.1 (0.94 - 1.3)	1.08 (0.81 - 1.45)
**Estonia**	5557 (5141 - 6027)	3475 (3189 - 3773)	5388 (4900 - 5924)	3567 (3247 - 3898)	377.87 (352.47 - 406.03)	197.88 (176.18 - 215.49)	195.55 (155.95 - 253.77)	112.91 (87.72 - 153.95)	6.8 (6.11 - 7.57)	5.69 (5 - 6.49)	3.63 (2.78 - 4.74)	3.17 (2.33 - 4.3)	0.84 (0.71 - 0.99)	0.87 (0.58 - 1.32)
**France**	2440 (2244 - 2641)	1088 (987 - 1201)	2304 (2086 - 2528)	1045 (938 - 1150)	81.99 (75.67 - 87.34)	38.34 (32.15 - 42.28)	54.7 (49.58 - 58.87)	26.07 (21.39 - 29.64)	3.36 (3.02 - 3.74)	3.52 (2.99 - 4.15)	2.37 (2.09 - 2.7)	2.49 (2.07 - 3.01)	1.05 (0.86 - 1.28)	1.05 (0.84 - 1.32)
**Italy**	2960 (2676 - 3264)	1434 (1282 - 1598)	2838 (2550 - 3152)	1339 (1196 - 1497)	104 (95.45 - 111.46)	56.58 (47.53 - 62.72)	73.61 (65.37 - 79.3)	41.07 (33.8 - 46.17)	3.51 (3.1 - 3.98)	3.95 (3.32 - 4.69)	2.59 (2.25 - 2.99)	3.07 (2.54 - 3.7)	1.12 (0.91 - 1.39)	1.18 (0.93 - 1.5)
**Malta**	2229 (2039 - 2433)	937 (813 - 1070)	2357 (2149 - 2580)	950 (840 - 1070)	170.75 (159.12 - 180.24)	98.35 (86.24 - 106.34)	118.13 (106 - 129.71)	68.64 (56.49 - 77.96)	7.66 (6.88 - 8.53)	10.49 (8.85 - 12.45)	5.01 (4.38 - 5.74)	7.22 (5.93 - 8.8)	1.37 (1.12 - 1.68)	1.44 (1.13 - 1.83)
**Slovenia**	4138 (3713 - 4623)	2345 (2065 - 2668)	3661 (3275 - 4104)	2191 (1923 - 2500)	122.64 (111.41 - 141.64)	66.26 (56.41 - 82.74)	84.7 (67.04 - 107.01)	40.81 (30.9 - 54.39)	2.96 (2.51 - 3.5)	2.83 (2.23 - 3.58)	2.31 (1.78 - 3.01)	1.86 (1.36 - 2.56)	0.95 (0.71 - 1.28)	0.81 (0.53 - 1.22)
**Moderate transition**														
**Croatia**	4439 (3962 - 4964)	2224 (1967 - 2522)	3932 (3472 - 4433)	2109 (1852 - 2394)	246.91 (230.74 - 261.03)	161.28 (143.89 - 173.21)	173.74 (142.52 - 207.96)	119.72 (95.31 - 144.01)	5.56 (4.89 - 6.33)	7.25 (6.22 - 8.46)	4.42 (3.53 - 5.53)	5.68 (4.46 - 7.22)	1.3 (1.07 - 1.6)	1.28 (0.92 - 1.79)
**Cyprus**	2084 (1897 - 2307)	864 (748 - 988)	2150 (1899 - 2456)	998 (877 - 1143)	206.36 (191.9 - 224.47)	120.03 (106.1 - 134.75)	118.5 (103.15 -140.65)	72.71 (61.02 - 88.38)	9.9 (8.73 - 11.23)	13.9 (11.57 - 16.7)	5.51 (4.49 - 6.76)	7.29 (5.79 - 9.17)	1.4 (1.12 - 1.76)	1.32 (0.97 - 1.8)
**Greece**	2320 (2132 - 2518)	1038 (941 - 1148)	2304 (2099- 2532)	1039 (933 - 1153)	143.12 (134.76 - 148.52)	100 (89.4 - 105.65)	113.14 (104.65 -119.46)	72.94 (63.17 - 78.96)	6.17 (5.6 - 6.79)	9.64 (8.47 - 10.96)	4.91 (4.38 - 5.51)	7.02 (6.03 - 8.17)	1.56 (1.33 - 1.83)	1.43 (1.18 - 1.73)

**Hungary**	5038 (4597 - 5538)	2674 (2431 - 2971)	4414 (3951 - 4966)	2462 (2199 - 2743)	302.06 (284.76 - 312.91)	185.57 (167.6 - 195.39)	221.55 (188.35 -258.48)	141.31 (116.76 -165.39)	6 (5.4 - 6.65)	6.94 (6.12 - 7.87)	5.02 (4.13 - 6.1)	5.74 (4.68 - 7.04)	1.16 (0.98 - 1.36)	1.14 (0.86 - 1.52)
**Latvia**	5014 (4593 - 5484)	2843 (2565 - 3155)	4763 (4314 - 5261)	2873 (2553 - 3214)	432.35 (409.78 - 457.83)	211.01 (192.04 -222.97)	270.95 (222.42 -330.45)	157.4 (127.39 -195.02)	8.62 (7.77 - 9.58)	7.42 (6.54 - 8.43)	5.69 (4.55 - 7.11)	5.48 (4.29 - 6.99)	0.86 (0.73 - 1.02)	0.96 (0.69 - 1.34)
**Lithuania**	4449 (4086 - 4872)	2702 (2431 - 3020)	4355 (3981- 4783)	2710 (2437 - 3028)	421.66 (399.08 - 439.7)	236.39 (213.78 -249.09)	297.18 (252.88 -348.59)	176.14 (144.67 -206.21)	9.48 (8.57 - 10.48)	8.75 (7.66 - 9.98)	6.82 (5.67 - 8.21)	6.5 (5.29 - 7.99)	0.92 (0.78 - 1.09)	0.95 (0.72 - 1.26)
**Poland**	3270 (2833 - 3775)	2027 (1726 - 2388)	2728 (2345 - 3162)	1686 (1430 - 2001)	252.43 (235.24 - 264.08)	143.1 (126.7 - 151.3)	174.68 (142.25 -211.15)	98.91 (78.99 - 120.18)	7.72 (6.61 - 9.01)	7.06 (5.87 - 8.49)	6.4 (5 - 8.2)	5.87 (4.49 - 7.67)	0.91 (0.72 - 1.16)	0.92 (0.63 - 1.33)
**Portugal**	1704 (1508 - 1931)	856 (743 - 986)	1864 (1636 - 2144)	890 (777 - 1013)	89.04 (83.07 - 93.33)	56.62 (49.88 - 60.87)	59 (54.09 - 63.31)	34.97 (29.38 - 38.97)	5.23 (4.56 - 5.99)	6.61 (5.57 - 7.86)	3.16 (2.71 - 3.7)	3.93 (3.25 - 4.76)	1.27 (1.02 - 1.58)	1.24 (0.97 - 1.59)
**Slovakia**	4175 (3748 - 4676)	2317 (2068 - 2621)	3664 (3261 - 4103)	2085 (1856 - 2353)	368.35 (346.06 - 381.28)	230.97 (207.6 - 243.98)	241.64 (196.24 -290.93)	166.75 (134.09 -201.34)	8.82 (7.82 - 9.96)	9.97 (8.64 - 11.5)	6.6 (5.26 - 8.28)	8 (6.33 - 10.11)	1.13 (0.94 - 1.36)	1.21 (0.87 - 1.69)
**Spain**	2459 (2266 - 2657)	1124 (1027 - 1232)	2351 (2145- 2588)	1113 (1002 - 1236)	94.12 (86.8 - 98.67)	49.72 (41.74 - 55.33)	60.71 (55.88 - 65.33)	31.98 (26.69 - 36.87)	3.83 (3.46 - 4.24)	4.43 (3.75 - 5.22)	2.58 (2.29 - 2.92)	2.87 (2.37 - 3.48)	1.16 (0.95 - 1.4)	1.11 (0.89 - 1.4)
**Weak transition**														
**Bulgaria**	4341 (3861 - 4893)	2423 (2163 - 2709)	4036 (3615 - 4523)	2254 (2002 - 2542)	412.06 (388.1 - 434.84)	256.36 (235.01 - 270.86)	300.9 (250.37 - 353.62)	190.23 (158.49 - 224.13)	9.49 (8.32 - 10.83)	10.58 (9.27 - 12.08)	7.46 (6.07 - 9.15)	8.44 (6.84 - 10.41)	1.11 (0.92 - 1.34)	1.13 (0.84 - 1.52)
**Romania**	4090 (3651 - 4610)	2379 (2114 - 2686)	3760 (3339 - 4248)	2196 (1962 - 2480)	299.11 (282.64 - 314.08)	207.85 (190.79- 219.46)	219.87 (187.44 - 257.9)	143.54 (119.71- 166.92)	7.31 (6.43 - 8.32)	8.74 (7.61 - 10.04)	5.85 (4.78 - 7.15)	6.54 (5.34 - 8)	1.19 (0.99 - 1.44)	1.12 (0.84 - 1.49)
**Overall**	91036 (82841 - 100234)	47311 (42455 - 52796)	84649 (76331 - 93992)	44872 (40041 - 50241)	5687.95 (5328.62 - 5991.66)	3290.96 (2932.92 - 3530.83)	3801.55 (3257.65 - 4393.35)	2270.78 (1866.09 - 2677.66)	6.25 (5.59 - 6.99)	6.96 (6.03 - 8.02)	4.49 (3.74 - 5.39)	5.06 (4.09 - 6.25)	1.11 (0.93 - 1.33)	1.13 (0.85 - 1.49)

**European Union average**	3372 (3068 - 3712)	1752 (1572 - 1955)	3135 (2827 - 3481)	1662 (1483 - 1861)	210.66 (197.36 - 221.91)	121.89 (108.63 - 130.77)	140.8 (120.65 - 162.72)	84.1 (69.11 - 99.17)	6.25 (5.59 - 6.99)	6.96 (6.03 - 8.02)	4.49 (3.74 - 5.39)	5.06 (4.09 - 6.25)	1.11 (0.93 - 1.33)	1.13 (0.85 - 1.49)

IHD= ischaemic heart disease. GBD 2019 definition describes IHD as International Classification of Disease (ICD) classes I20-25.9, namely: angina pectoris, acute myocardial infarction, subsequent ST elevation (STEMI) and non-ST elevation (NSTEMI) myocardial infarction, certain current complications following ST elevation (STEMI) and non-ST elevation (NSTEMI) myocardial infarction (within the 28 day period), other acute ischaemic heart disease, and chronic ischaemic heart disease.
Data and definitions from Global Burden of Disease Database, 2019

REFERENCES

1. European Commission: Directorate-General for R, Innovation, Prevost S, et al. Transitions performance index 2021 – Towards fair and prosperous sustainability: Publications Office of the European Union; 2022.

2. Vogel B, Acevedo M, Appelman Y, et al. The Lancet women and cardiovascular disease Commission: reducing the global burden by 2030. *Lancet* 2021; **397**(10292): 2385-438.

3. Woodward M. Cardiovascular Disease and the Female Disadvantage. *Int J Environ Res Public Health* 2019; **16**(7).

4. Zhu D, Chung HF, Dobson AJ, et al. Age at natural menopause and risk of incident cardiovascular disease: a pooled analysis of individual patient data. *Lancet Public Health* 2019; **4**(11): e553-e64.

5. Mishra SR, Chung HF, Waller M, et al. Association Between Reproductive Life Span and Incident Nonfatal Cardiovascular Disease: A Pooled Analysis of Individual Patient Data From 12 Studies. *JAMA Cardiol* 2020; **5**(12): 1410-8.

6. Elder P, Sharma G, Gulati M, Michos ED. Identification of female-specific risk enhancers throughout the lifespan of women to improve cardiovascular disease prevention. *Am J Prev Cardiol* 2020; **2**: 100028.

7. Maas A, Rosano G, Cifkova R, et al. Cardiovascular health after menopause transition, pregnancy disorders, and other gynaecologic conditions: a consensus document from European cardiologists, gynaecologists, and endocrinologists. *Eur Heart J* 2021; **42**(10): 967-84.

8. Roeters van Lennep JE, Tokgözoğlu LS, Badimon L, et al. Women, lipids, and atherosclerotic cardiovascular disease: a call to action from the European Atherosclerosis Society. *Eur Heart J* 2023; **44**(39): 4157-73.

9. Xiao B, Velez Edwards DR, Lucas A, et al. Inference of Causal Relationships Between Genetic Risk Factors for Cardiometabolic Phenotypes and Female-Specific Health Conditions. *J Am Heart Assoc* 2023; **12**(5): e026561.

10. Dong L, Teh DBL, Kennedy BK, Huang Z. Unraveling female reproductive senescence to enhance healthy longevity. *Cell Res* 2023; **33**(1): 11-29.

11. Lu D, Valdimarsdóttir UA, Wei D, et al. Perinatal depression and risk of maternal cardiovascular disease: a Swedish nationwide study. *Eur Heart J* 2024; **45**(31): 2865-75.

12. Vaccarino V, Bremner JD. Stress and cardiovascular disease: an update. *Nat Rev Cardiol* 2024; **21**(9): 603-16.

13. Ebong IA, Quesada O, Fonkoue IT, et al. The Role of Psychosocial Stress on Cardiovascular Disease in Women: JACC State-of-the-Art Review. *J Am Coll Cardiol* 2024; **84**(3): 298-314.

14. Acevedo-Rodriguez A, Kauffman AS, Cherrington BD, Borges CS, Roepke TA, Laconi M. Emerging insights into hypothalamic-pituitary-gonadal axis regulation and interaction with stress signalling. *J Neuroendocrinol* 2018; **30**(10): e12590.

15. Oyola MG, Handa RJ. Hypothalamic-pituitary-adrenal and hypothalamic-pituitary-gonadal axes: sex differences in regulation of stress responsivity. *Stress* 2017; **20**(5): 476-94.

16. Sardinha L, Maheu-Giroux M, Stockl H, Meyer SR, Garcia-Moreno C. Global, regional, and national prevalence estimates of physical or sexual, or both, intimate partner violence against women in 2018. *Lancet* 2022; **399**(10327): 803-13.

17. Chandan JS, Thomas T, Bradbury-Jones C, Taylor J, Bandyopadhyay S, Nirantharakumar K. Risk of Cardiometabolic Disease and All-Cause Mortality in Female Survivors of Domestic Abuse. *J Am Heart Assoc* 2020; **9**(4): e014580.

18. Stubbs A, Szoeke C. The Effect of Intimate Partner Violence on the Physical Health and Health-Related Behaviors of Women: A Systematic Review of the Literature. *Trauma Violence Abuse* 2022; **23**(4): 1157-72.

19. Lyons JG, Cauley JA, Fredman L. The Effect of Transitions in Caregiving Status and Intensity on Perceived Stress Among 992 Female Caregivers and Noncaregivers. *J Gerontol A Biol Sci Med Sci* 2015; **70**(8): 1018-23.

20. Suglia SF, Koenen KC, Boynton-Jarrett R, et al. Childhood and Adolescent Adversity and Cardiometabolic Outcomes: A Scientific Statement From the American Heart Association. *Circulation* 2018; **137**(5): e15-e28.

21. Garad Y, Maximova K, MacKinnon N, McGrath JJ, Kozyrskyj AL, Colman I. Sex-Specific Differences in the Association Between Childhood Adversity and Cardiovascular Disease in Adulthood: Evidence From a National Cohort Study. *Can J Cardiol* 2017; **33**(8): 1013-9.

22. Rohleder N, Schommer NC, Hellhammer DH, Engel R, Kirschbaum C. Sex differences in glucocorticoid sensitivity of proinflammatory cytokine production after psychosocial stress. *Psychosom Med* 2001; **63**(6): 966-72.

23. Samad Z, Boyle S, Ersboll M, et al. Sex differences in platelet reactivity and cardiovascular and psychological response to mental stress in patients with stable ischemic heart disease: insights from the REMIT study. *J Am Coll Cardiol* 2014; **64**(16): 1669-78.

24. Mehta PK, Hermel M, Nelson MD, et al. Mental stress peripheral vascular reactivity is elevated in women with coronary vascular dysfunction: Results from the NHLBI-sponsored Cardiac Autonomic Nervous System (CANS) study. *Int J Cardiol* 2018; **251**: 8-13.

25. Vaccarino V, Sullivan S, Hammadah M, et al. Mental Stress-Induced-Myocardial Ischemia in Young Patients With Recent Myocardial Infarction: Sex Differences and Mechanisms. *Circulation* 2018; **137**(8): 794-805.

26. Lyon AR, Citro R, Schneider B, et al. Pathophysiology of Takotsubo Syndrome: JACC State-of-the-Art Review. *J Am Coll Cardiol* 2021; **77**(7): 902-21.

27. Sullivan S, Young A, Hammadah M, et al. Sex differences in the inflammatory response to stress and risk of adverse cardiovascular outcomes among patients with coronary heart disease. *Brain Behav Immun* 2020; **90**: 294-302.

28. Sullivan S, Hammadah M, Wilmot K, et al. Young Women With Coronary Artery Disease Exhibit Higher Concentrations of Interleukin-6 at Baseline and in Response to Mental Stress. *J Am Heart Assoc* 2018; **7**(23): e010329.

29. Hernandez MF, Rodriguez F. Health Techequity: Opportunities for Digital Health Innovations to Improve Equity and Diversity in Cardiovascular Care. *Curr Cardiovasc Risk Rep* 2023; **17**(1): 1-20.

30. Gray R, Indraratna P, Lovell N, Ooi SY. Digital health technology in the prevention of heart failure and coronary artery disease. *Cardiovasc Digit Health J* 2022; **3**(6 Suppl): S9-s16.

31. Azizi Z, Adedinsewo D, Rodriguez F, Lewey J, Merchant RM, Brewer LC. Leveraging Digital Health to Improve the Cardiovascular Health of Women. *Curr Cardiovasc Risk Rep* 2023; **17**(11): 205-14.

32. Morales-Lara AC, Garzon-Siatoya WT, Fernandez-Campos BA, Adedinsewo D. Advancing Our Understanding of Women's Cardiovascular Health Through Digital Health and Artificial Intelligence. *JACC Adv* 2023; **2**(2): 100272.

33. Cai Y, Cai YQ, Tang LY, et al. Artificial intelligence in the risk prediction models of cardiovascular disease and development of an independent validation screening tool: a systematic review. *BMC Med* 2024; **22**(1): 56.

34. Huerne K, Eisenberg MJ. Advancing telemedicine in cardiology: A comprehensive review of evolving practices and outcomes in a postpandemic context. *Cardiovasc Digit Health J* 2024; **5**(2): 96-110.
